# MicroRNA-124-3p inhibits cell growth and metastasis in cervical cancer by targeting IGF2BP1

**DOI:** 10.3892/etm.2022.11157

**Published:** 2022-01-20

**Authors:** Ping Wang, Li Zhang, Junhui Zhang, Gangshu Xu

Exp Ther Med 15:1385–1393, 2018; DOI: 10.3892/etm.2017.5528

Following the publication of this paper, it was drawn to the Editors’ attention by a concerned reader that the Transwell cell migration assay data shown in Figs. 2D and 6D were strikingly similar to data appearing in different form in other articles by different authors. Owing to the fact that the contentious data in the above article had already been published elsewhere, or were already under consideration for publication, prior to its submission to *Experimental and Therapeutic Medicine*, the Editor has decided that this paper should be retracted from the Journal. The authors were asked for an explanation to account for these concerns, but the Editorial Office did not receive any reply. The Editor apologizes to the readership for any inconvenience caused.

